# Problematic internet use profiles and psychosocial risk among adolescents

**DOI:** 10.1371/journal.pone.0257329

**Published:** 2021-09-14

**Authors:** Halley M. Pontes, Mirna Macur

**Affiliations:** 1 Department of Organizational Psychology, Birkbeck, University of London, London, United Kingdom; 2 Angela Boškin Faculty of Health Care, Jesenice, Slovenia; National Cheng Kung University College of Medicine, TAIWAN

## Abstract

**Objective:**

Although Problematic Internet Use (PIU) is an emerging area of study in psychology, little is known about the unique features of specific subgroups of internet users and their psychosocial vulnerabilities within robust and nationwide populations.

**Methods:**

The aim of this study was to identify distinct latent groups of internet users based on their PIU risk and to compare their psychosocial outcomes. To achieve this, a nationally representative sample of adolescents of the same grade (*N* = 1,066, Mean_age_ = 13.46 years, range = 12–16) was recruited from several schools in Slovenia through stratified random sampling.

**Results:**

A Latent Profile Analysis (LPA) revealed a two-class solution, with Class 1 (*n* = 853, 80%) featuring ‘*low PIU risk*’ participants and Class 2 (*n* = 213, 20%) including ‘*high PIU risk*’ participants. Behaviorally, the main feature of Class 1 denoted ‘*time management difficulties*’ while Class 2 was best characterized by ‘*mood and time management issues*’. Further frequentist and Bayesian analyses indicated that Class 2 presented greater psychosocial risk compared to Class 1 due to significantly higher levels of PIU (generalized and across specific PIU subfactors) coupled with lower levels of subjective well-being and self-control.

**Conclusions:**

Contrary to what was initially envisaged, the two classes did not differ in terms of perceived quality in parent-child relationship. This study shows that PIU patterns and symptom-severity may be developmentally specific, further highlighting the need for clinically age-adjusted PIU screening practices within epidemiological and healthcare settings.

## Introduction

The latest statistics suggest that approximately 4.66 billion individuals worldwide actively use the internet, representing a total global penetration rate of 59% [1]. Given the multiple functionalities and services offered by the internet as a contemporary leisure and professional tool, its use has been found to lead to several psychosocial benefits [2], with social media use constituting a constructive coping strategy capable of helping internet users manage their loneliness and anxiety levels during unprecedented pandemic times [3], and healthy forms (e.g., harmonious as opposed to obsessive passion) of online gaming engagement being associated with increased bonding capital, decreased loneliness, and enhanced well-being [4].

Theoretically, these benefits can be understood within the Needs-Affordances-Features perspective [5] on technology (e.g., internet) use, which suggests that users’ psychological needs drive their usage levels of specific digital technologies and features (e.g., social media, gaming, gambling, emailing, pornography, shopping, etc.), to the extent that such usage provides salient affordances that ultimately contribute to satisfying their basic psychological needs.

Notwithstanding this, the perils associated to excessive and dysregulated use of the internet have been widely documented within the psychological literature [6], with the first studies on Problematic Internet Use (PIU), also controversially termed as ‘Internet addiction’ [7,8], being published nearly 25 years ago [see 9,10]. Although, generalized PIU is not currently recognized as an official mental health disorder, gaming disorder [seen as a specific form of PIU, see 11], has been recently recognized as a *bona fide* addictive disorder [12], and previously proposed as a tentative disorder by the American Psychiatric Association (APA) under the term of ‘Internet Gaming Disorder’ [13].

In relation to the epidemiological status of PIU, a number of key review studies reported important findings. A recent meta-analytic study reviewing 113 studies from 31 countries suggested a prevalence rate of PIU of 7%, with prevalence increasing over time and differing according to the assessment tool utilized [14]. In terms of gender-based prevalence, a recent meta-analysis of 101 studies from 34 nations [15] reported that male internet users presented with higher PIU tendency compared to females, with the largest effect sizes in gender-based prevalence estimates being observed in Asia and the lowest in North America. As for geographical prevalence estimates, a meta-analysis of 80 studies from 31 countries covering seven world regions found a global prevalence of PIU of 6%, with the highest prevalence being observed in the Middle East region (10.9%), followed by North America (8%), and Southern and Eastern Europe (6.1%). Although epidemiology appears to vary across different regions, PIU vulnerability is relatively equivalent across Western and non-Western nations [14].

Several studies have also widely documented the detrimental psychosocial impacts of PIU. A recent study investigating cognitive performance in PIU concluded that the existing evidence supports a significant association between PIU and decrements across a range of neuropsychological domains (e.g., inhibitory control, decision-making, and working memory) [16]. Further investigation indicated that PIU may result in sleep problems (i.e., decreased sleep quality and duration) [17] and decreased perceived social support [18].

Recently, Fumero, Marrero [19] have investigated the role specific PIU risk factors linked to psychopathology (e.g., depression and anxiety), personality (e.g., aggressiveness, hostility, and sensation seeking self-esteem), and social factors (e.g., peer problems and family difficulties), and found that emotional personal factors (e.g., hostility, depression, and anxiety) had greater associations with PIU than social factors, with PIU being mainly associated with mood disturbances. In terms of comorbidities, PIU has been linked to greater levels of somatic symptoms, clinical depression, psychoticism, paranoid ideation, serious mental illness, and suicidality (i.e., ideations, plans, and attempts) [20], with additional research supporting a consistent association between PIU and a wide range of adverse mental health outcomes such as attention deficit/hyperactivity disorder [21], social anxiety [22], and alcohol abuse [23].

Despite its current unofficial clinical status, PIU affects a significant, albeit relatively small, portion of the population worldwide, further warranting additional scientific scrutiny on its potential impacts, especially within large-scale and nationwide studies that are able to provide more generalizable estimates regarding the pitfalls of PIU.

### The present study

The main aim of the present study was twofold: (i) to identify and characterize unique subgroups of internet users based on their PIU risk in order to further (ii) compare their distinct psychosocial differences. To achieve this, we have opted to conduct a Latent Profile Analysis (LPA), because this analytical approach is usually employed to understand variables (or indicators) that can predict high-risk behaviors through identification of latent subgroups presenting varying risk levels [24,25].

This statistical approach offers important advantages over more traditional dimensional approaches (e.g., factor analytical) because it does not assume that the probability of a response on a putative variable is a linear function of a latent factor, instead, the variables used in the LPA predict class membership based on the intersection of variables used in the model defining subgroups that differ in qualitative terms [26]. Ultimately, this investigatory approach aligns with the latest recommendations to investigate problematic technology use behaviors employing non-confirmatory approaches [27]. Thus, in light of the existing literature and the aim established for this study, the two following exploratory working hypotheses were devised:

Hypothesis 1 (H1): specific latent subgroups among internet users will be identified based on their unique PIU patterns and risk. We expect that a unique typology of participants will emerge based on their intricate shared PIU risks.

Hypothesis 2 (H2): the latent subgroups of internet users identified in the LPA will differ in terms of their psychosocial risks whereby, when compared to participants showing lower risk, those experiencing higher risk will be expected to exhibit greater levels of PIU symptoms coupled with reduced levels of subjective well-being, quality in perceived parent-child relationship, and self-control levels.

Given the large amount of PIU studies using relatively low sample sizes recruited through convenience sampling, this study will contribute to the field by further helping build the knowledge base regarding the psychosocial risks associated with PIU within a large-scale and nationwide framework, generating findings that better reflect users’ experiences and psychosocial vulnerabilities.

## Material and methods

### Participants

Data for this study was collected nationwide in the context of a large epidemiological study in Slovenia. Participants were adolescents recruited via stratified random sampling using all primary school classes (i.e., eighth grade) as the primary sampling units. The stratification was based on the twelve statistical regions of Slovenia according to the Nomenclature of Territorial Units for Statistics (i.e., NUTS 3) and population density.

All school principals of the randomly sampled schools were contacted and invited to participate in the study. The study procedures were carried out in accordance with the Declaration of Helsinki. The Institutional Review Board of Nottingham Trent University (United Kingdom) and the National Institute of Public Health (Slovenia) approved the study. All subjects were informed about the study and they all provided written informed consent. Parental written informed consent was provided by parents and/or legal guardians of participants aged below 15 years.

A computer-assisted survey containing all main measures of the study was administered by trained staff and electronically completed by all participants in the school setting (April to May 2015). Prior to the data collection, informed consent was obtained from all parents and/or legal guardians, and a sample of 1,095 participants was initially recruited to the present study. All participants presenting with severe missing values (i.e., ≥ 10%, *n* = 24, 2.2%) and carelessness or inattentiveness when filling out the survey (i.e., ≥ 50% response invariability, *n* = 5, 0.5%) were excluded. Thus, the effective sample comprised 1,066 adolescents (*Mean*_*age*_ = 13.46 years, *SD*_*age*_ = 0.58) (see Table 1).

**Table 1:** Main sociodemographic characteristics of the sample (N = 1,066).

**Sociodemographic features**	
Age (years) (mean, *SD*)	13.46 (0.58)
Gender (female) (*n*, %)	533 (50)
**Age distribution** (*n*, %)	
12-year-olds	19 (1.78)
13-year-olds	566 (53.10)
14-year-olds	456 (42.78)
15-year-olds	23 (2.16)
16-year-olds	2 (0.18)
**Geographic Region** (*n*, %)^a^	
Pomurska	138 (5.61)
Podravska	242 (14.42)
Koroška	45 (3.79)
Savinjska	81 (12.68)
Zasavska	57 (2.59)
Posavska	22 (3.81)
Jugovzhodna Slovenija	86 (7.41)
Osrednjeslovenska	227 (26.21)
Gorenjska	104 (10.53)
Primorsko-notranjska	20 (2.51)
Goriška	23 (5.92)
Obalno-kraška	21 (4.58)
**Internet use behaviors** (hours) (mean, *SD*)	
Daily time spent on the internet on weekdays for leisure	2.87 (2.55)
Daily time spent on the internet on weekdays for leisure through mobile phone	1.75 (2.40)
Daily time spent on the internet on weekends for leisure	3.34 (2.87)
Daily time spent on the internet on weekends for leisure through mobile phone	1.99 (2.44)

*Note*. Estimates were computed using sampling weights unless otherwise specified. SD = standard deviation.

^a^ Percentages do not add up to 100% due to rounding inaccuracies.

### Measures

#### Sociodemographic and internet use data

Sociodemographic data were collected on participants’ age, gender, and region of residence. Moreover, internet use behaviors included assessing participants daily time spent on the internet for leisure purposes (i.e., number of hours) on weekdays and weekends in general and through their mobile phones more specifically.

#### Problematic internet use

The Problematic Internet Use Questionnaire Short-Form (PIUQ-SF-6) [28] was used to assess PIU. This brief tool conceptualizes PIU according to three subfactors: Obsession (items 2: “*How often do you feel tense*, *irritated*, *or stressed if you cannot use the Internet for as long as you want to*?” and 6: “*How often does it happen to you that you feel depressed*, *moody*, *or nervous when you are not on the Internet and these feelings stop once you are back online*?”), Neglect (items 1: “*How often do you spend time online when you’d rather sleep*?” and 5: “*How often do people in your life complain about you spending too much time online*?”), and Control Disorder (items 3: “*How often does it happen that you wish to decrease the amount of time spend online but you do not succeed*?” and 4: “*How often do you try to conceal the amount of time spent online*?”). Each factor contains two items rated on a five-point Likert scale ranging from ‘never’ to ‘always/almost always’ and total obtainable scores can range from 6 to 30, with higher scores indicating greater levels of PIU. The PIUQ-SF-6 has been previously shown to be psychometrically sound and has been utilized across several studies [e.g., 29–35]. In the present study, the PIUQ-SF-6 exhibited excellent internal consistency levels (Cronbach’s α = .84; McDonald’s ω = .90).

#### Subjective well-being

Participants’ subjective well-being was evaluated using a composite measure comprising the following two Likert-type self-report items: “*Are you satisfied with your life*?” (rated from 1 = ‘extremely dissatisfied’ to 10 = ‘extremely satisfied’) and “*In general*, *how would you consider your mental health*?” (rated from 1 = ‘bad’ to 5 = ‘excellent’). The two items were summed to represent an overall measure of participants’ subjective well-being, with higher scores indicating increased levels of perceived subjective well-being. In the present study, this composite measure exhibited excellent internal consistency levels (Cronbach’s α = .67; McDonald’s ω = .75).

#### Parent-child relationship

The quality of parental relationship was assessed using four items measuring perceived levels of relational affection between the participants and their parents (e.g., “*My parents and I try to spend much time together*”). These four items have been previously used in similar research that supported its psychometric properties [36]. Moreover, each item represents different shared activities and levels of emotional closeness that are rated on a five-point Likert scale ranging from 1 = ‘very untrue’ to 5 = ‘very true’, with higher scores reflecting increased perceived quality in parental relationship. In the present study, this measure has exhibited excellent levels of internal consistency (Cronbach’s α = .85; McDonald’s ω = .87).

#### Self-control

Participants’ level of self-control was evaluated using a brief seven-item measure that has been employed in previous PIU research supporting its adequate psychometric properties [36]. All items of this psychometric test (e.g., “*I feel like I am a ticking time bomb*”) are rated on a five-point Likert scale ranging from 1 = ‘strongly agree’ to 5 = ‘strongly disagree’, with higher scores indicating greater levels of self-control. In the present study, this measure has exhibited excellent internal consistency levels (Cronbach’s α = .82; McDonald’s ω = .86).

### Statistical analysis

All statistical analyses were performed using the statistical packages R version 4.0.3 “Bunny-Wunnies Freak Out” [37] and M*plus* version 8.2 [38]. The analytical approach adopted in this study included the three main steps: (i) basic descriptive statistical analysis (e.g., means, standard deviations) of the samples’ main features, (ii) LPA to identify specific typologies and latent subgroups (i.e., classes) of internet users based on their PIU risk, and (iii) inferential statistics (e.g., frequentist and Bayesian independent samples Welch’s *t*-tests) accompanied by effect size estimations (e.g., Cohen’s *d*) to better characterize the latent classes identified in the LPA.

While LPA was conducted with M*plus* due to its ability to incorporate sampling weights in the mixture modeling procedure, all remaining analyses were performed on R using the packages *careless* version 1.1.3 [39], *BayesFactor* version 0.9.12–4.2 [40], *psych* version 2.0.9 [41], and *weights* version 1.0.1 [42] embedding sampling weights in the estimations. The estimated Bayes factors (BF_10_) were interpreted according to the recommendations provided by Lee and Wagenmakers [43] and Cohen’s *d* effect sizes were interpreted as small (*d* = .2), medium (*d* = .5), and large (*d* = .8) [44].

The LPA is a mixture modeling statistical analysis that can be used to identify groups of individuals that may be similar in their response patterns to specific variables. In the presented study, LPA was conducted using all six PIUQ-SF-6 items (continuous variables) as opposed to using the three subfactors of the PIUQ-SF-6 since this approach offers enhanced analytical granularity in the description and interpretation of the latent classes due to the greater amount of variables informing the estimation procedure.

To determine the optimal number of latent classes to be extracted in the LPA, the following three Information Theory Fit Indices were inspected: Akaike’s Information Criterion (AIC) [45], Bayesian Information Criterion (BIC) [46], and the Sample-Size Adjusted Bayesian Information Criterion (SSABIC) [47], with lower values indicating better model fit [48]. Additionally, Entropy [49] was computed to ascertain whether the estimated solution was optimal. Entropy ranges from 0 to 1, and larger values suggest greater clarity in the identification of the latent classes while values approaching 1 indicate clear separation of the classes [50]. Furthermore, the adjusted Lo-Mendell-Rubin Likelihood Ratio Test (L-M-R Test) [51] was also computed to determine the optimal number of classes by using its *p*-value, with low and significant *p*-values (i.e., *p* < .05) suggesting that the K_0_-class model has significantly better fit to the data than the K-_1_-class model [48]. This likelihood-based test performs less well compared to the Bootstrap Likelihood Ratio Test (BLRT) [52], however, the BLRT cannot be computed when using sampling weights.

Finally, after the optimal number of latent classes underlying the data was identified, further comparative analyses were conducted to characterize the unique features of each class in relation to overall and specific PIU, subjective well-being, parent-child relationship, and self-control levels.

## Results

### Descriptive statistics

In terms of basic weighted sociodemographic features, the mean age of the sample was 13.46 years (*SD*_*age*_ = 0.58, range = 12–16 years) and the gender distribution was even (*n* = 533, 50% female). Table 1 provides a summary of all key sociodemographic features, including the sample’s residency statistics. In relation to internet use behaviors, participants reported spending greater amounts of time on the internet for leisure purposes on the weekends (Mean = 3.34 hours, SD = 2.87), with mobile internet use being mostly prominent on the weekends (Mean = 1.99 hours, SD = 2.44).

### LPA

#### Determining the number of latent classes

The LPA was performed using specified starting values with random starts (2,000 random sets of starting values for the initial stage and 20 final stage optimizations) to avoid solutions at local maxima, using maximum likelihood estimation with robust standard errors (MLR) as the estimator. The full results of the LPA for models with 1 through 4 classes are presented in Table 2. After evaluating all four models, the two-class model was chosen since it provided the best fit to the data.

**Table 2:** Model fit of the latent profile analysis models from 1 through 4 classes (N = 1,066).

Number of latent classes	BIC	AIC	SSABIC	Entropy	L-M-R Test	L-M-R Test, *p*-value
1	19665.320	19605.660	19627.206	-	-	-
**2**	**17636.631**	**17542.169**	**17576.284**	**0.935**	**2035.776**	**< 0.001**
3	17108.649	16979.385	17026.068	0.902	565.203	0.4523
4	15954.033	15789.968	15849.219	1.000	1179.253	0.1302

*Note*. Estimates were computed using sampling weights unless otherwise specified. The chosen model is highlighted in bold in the table. BIC = Bayesian Information Criterion; AIC = Akaike’s Information Criterion; SSABIC = Sample-Size Adjusted Bayesian Information Criterion; L-M-R Test = Lo-Mendell-Rubin Adjusted Likelihood Ratio Test.

The decision to favor the two-class model was based on the fact that the L-M-R Test was not statistically significant for the three- (*p* = .45) or four-class (*p* = .13) models, with entropy being optimal for the two-class model (i.e., 0.935). Furthermore, the two-class model was able to correctly classify 98.8% of Class 1 and 95.4% of Class 2 participants, which is well-above the recommended threshold of .70 [53]. Although the three- and four-class models had lower BIC, AIC, and SSABIC values, the three-class model was disregarded as it presented with (i) less optimal L-M-R Test *p*-values (i.e., > .05), (ii) worse accuracy in class membership assignment due to lower entropy levels, and (iii) less theoretical clarity in interpreting the solution derived. Based on current LPA conventions [54], the alternative four-class model was also disregarded as it did not converge.

#### Latent class characterization and interpretation

**Fig 1:**
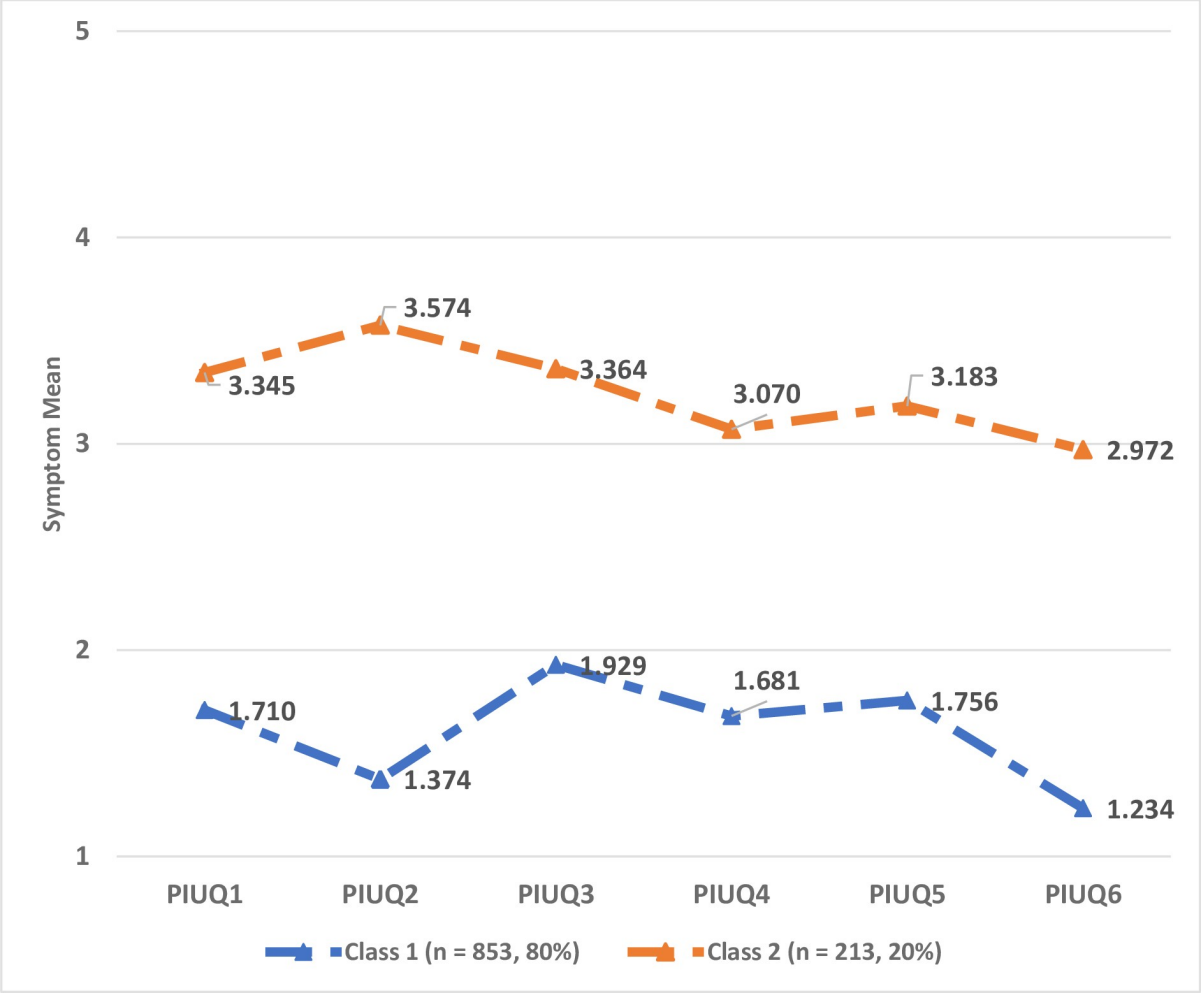
Characterization of the two latent classes of problematic internet users obtained from the Latent Profile Analysis (N = 1,066). *Note*. Problematic internet use symptom means for each item of the Problematic Internet Use Questionnaire Short-Form (PIUQ-SF-6). PIUQ1 = *How often do you spend time online when you’d rather sleep*?, PIUQ2 = *How often do you feel tense*, *irritated*, *or stressed if you cannot use the Internet for as long as you want to*?; PIUQ3 = *How often does it happen that you wish to decrease the amount of time spent online but you do not succeed*?; PIUQ4 = *How often do you try to conceal the amount of time spent online*?; PIUQ5 = *How often do people in your life complain about you spending too much time online*?; PIUQ6 = *How often does it happen to you that you feel depressed*, *moody*, *or nervous when you are not on the Internet and these feelings stop once you are back online*?.

The two final classes that emerged from the LPA alongside their main characteristics in terms of response patterns to the PIUQ-SF-6 items (i.e., mean PIU severity on each item) is illustrated in Fig 1. The two classes were labelled relative to their problematic usage risk in order to add qualitative value to the interpretability of the LPA results as commonly done in similar studies [e.g., 55–59]. Overall, Class 1 (*n* = 853, 80%) presented lower PIU symptom-severity in comparison to Class 2 (*n* = 213, 20%). Thus, Class 1 was labelled as ‘*low PIU risk*’ while Class 2 was labelled as ‘*high PIU risk*’.

In terms of class characterization, ‘*low PIU risk*’ participants presented particularly high symptoms related to the PIU subfactors associated with Control Disorder (item 3) and Neglect (items 5 and 1), clearly indicating that the main behavioral feature of this class is marked by time management difficulties. In contrast, ‘*high PIU risk*’ participants exhibited elevated symptoms related to the Obsession (item 2), Control Disorder (item 3), and Neglect (item 1) subfactors, suggesting that the main behavioral aspect of this class is marked by mood and time management issues.

#### Cross-class comparisons

In addition to the aforementioned procedures, the two classes were further compared against each other with respect to participants’ overall levels of PIU and its three specific subfactors, alongside subjective well-being, parent-child relationship, and self-control levels (see Table 3).

**Table 3:** Cross-class comparisons of high and low risk of problematic internet users for the two latent classes (N = 1,066).

Variable	Latent Class		Frequentist		Bayesian		Effect Size
	Low PIU risk	High PIU risk	Welch’s *t* (*df*)	*p*-value	BF_10_	Bayes Classification	Cohen’s *d*
	(*n* = 853, 80%)	(*n* = 213, 20%)					
Problematic internet use (mean)	10.61	15.64	-12.24 (275.48)	< .001	67	Very strong evidence for H_1_	1.05
*Obsession* (mean)	3.00	4.92	-11.07 (255.38)	< .001	55	Very strong evidence for H_1_	1.06
*Neglect* (mean)	3.74	5.38	-10.25 (278.95)	< .001	47	Very strong evidence for H_1_	0.84
*Control Disorder* (mean)	3.63	5.00	-7.64 (287.60)	< .001	25	Strong evidence for H_1_	0.66
Subjective well-being (mean)	12.02	11.35	2.91 (309.36)	.004	1.70	Anecdotal evidence for H_1_	0.28
Parent-child relationship (mean)	15.68	15.21	1.79 (330.10)	.074	0.89	Anecdotal evidence for H_0_	0.14
Self-control (mean)	23.35	15.21	25.51 (596.09)	< .001	> 100	Extreme evidence for H_1_	0.24

*Note*. Estimates were computed using sampling weights. Low PIU risk (*n* = 857, 80%) and High PIU class (*n* = 214, 20%). Estimates were conducted with sampling weights. PIU = problematic internet use; t = Welch’s *t*-test statistic; df = degrees of freedom; SD = standard deviation; BF_10_ = Bayes factor; H_0_ = null hypothesis; H_1_ = alternative hypothesis. Bayes classification for BF_10_ based on > 100, extreme evidence for H_1_; 30–100, very strong evidence for H_1_; 10–30, strong evidence for H_1_; 3–10 moderate evidence for H_1_; 1–3 anecdotal evidence for H_1_; 1, no evidence; 1–0.33, anecdotal evidence for H_0_; 0.33–0.10, moderate evidence for H_0_; 0.10–0.03, strong evidence for H_0_; 0.03–0.01, very strong evidence for H_0_; <0.01, extreme evidence for H_0_.

Accordingly, in comparison to the ‘*low PIU risk*’ participants (i.e., Class 1), participants classed as having ‘*high PIU risk*’ (i.e., Class 2) presented with significantly higher overall PIU symptoms (*t*_[275.48]_ = -12.24, *p* < .001; BF_10_ = 67, *d* = 1.05) and across the three PIU subfactors: Obsession (*t*_[255.38]_ = -11.07, *p* < .001; BF_10_ = 55, *d* = 1.06), Neglect (*t*_[278.95]_ = -10.25, *p* < .001; BF_10_ = 47, *d* = 0.84), and Control Disorder (*t*_[287.60]_ = -7.64, *p* < .001; BF_10_ = 25, *d* = 0.66).

Additionally, as expected, in comparison to ‘*low PIU risk*’ participants, those with membership to the ‘*high PIU risk*’ class exhibited significantly lower levels of subjective well-being (*t*_[309.36]_ = 2.91, *p* = .004; BF_10_ = 1.70, *d* = 0.28) and self-control (*t*_[596.09]_ = 25.51, *p* < .001; BF_10_ > 100, *d* = 0.24). Contrary to what has been hypothesized, parent-child relationship did not differ significantly between the two classes (*t*_[330.10]_ = 1.79, *p* = .074; BF_10_ = 0.89, *d* = 0.14). The magnitude of the observed effect sizes across the means compared ranged from small to large. Taken together, these results lend support to H1, and partially support H2.

## Discussion

The main aim of this study was to help advance the literature on PIU by exploring its psychosocial risks in a nationally representative sample of adolescents. This constitutes an important goal given that most previous research on PIU focused on investigating older populations (e.g., emerging adults and adults). This study allowed us to empirically identify and characterize unique subgroups of internet users based on their unique PIU risk so that we could estimate their potential psychosocial risks based on group membership. Below, we discuss the implications of the findings obtained according to the existing literature and the goals of the present study.

In the context of H1, we expected that specific subgroups of adolescent internet users would emerge based on their PIU risk. Although previous research has performed Latent Class Analysis (LCA) to investigate this issue, these studies have employed different assessment methodologies using the existing alternative forms of the PIUQ assessment tool [60]. To the best of our knowledge, only one study has utilized the PIUQ-SF-6 to identify latent classes [28].

Using a nationally representative sample of Hungarian adolescents (mean age of 16.4 years), Demetrovics, Király (28) also found two distinct latent classes based on the three subfactors of the PIUQ-SF-6. More specifically, their ‘*at risk of PIU*’ class (comprising 14.4% of the sample) was mainly characterized by elevations on Neglect (items 1 and 5), Obsession (items 2 and 6), and Control Disorder (items 3 and 4). Based on the LPA findings of our study, ‘*high PIU risk*’ participants exhibited a slightly different (but still comparable) pattern of PIU risk as this class was mainly characterized by the one item of the Obsession (i.e., item 2), Control Disorder (i.e., item 3), and the Neglect (i.e., item 1) subscales.

While ‘*at risk of PIU*’ participants from the study conducted by Demetrovics, Király [28] were mainly characterized in terms of the symptoms measured by items 1 and 5, in our study the most pronounced features of the ‘*high PIU risk*’ class related to symptoms measured by items 1, 2, and 3. Although there are some overlapping features between these two groups (i.e., item 1), our ‘*high PIU risk*’ class was proportionally larger (i.e., 20%) and experienced more diffuse PIU symptoms beyond the Neglect subfactor.

This nuanced and seemingly discrepant finding might underlie unique developmental features and parental mediation approaches adopted by parents to regulate their child’s internet use behaviors. It is known that parental mediation is a dynamic process whereby parents adapt their mediation styles to their developing child while also adjusting their internet mediation styles to their child’s stage of development [61,62]. Thus, it is plausible that internet-related parental mediation strategies may have differed between children and adolescent internet users from our study and the study conducted by Demetrovics, Király [28].

We note that although parents may employ developmentally-adjusted mediation strategies when regulating their child’s internet use, there is currently no robust evidence supporting a strong link between specific parental mediation strategies and decreased PIU levels [63]. However, it is likely that different mediation strategies may lead to distinct outcomes in relation to internet use behavior and its many resulting effects. Relatedly, psychoeducational interventions may also be fruitful in raising awareness about the detrimental effects of PIU by fostering healthy and judicious usage of the internet, preventing problematic usage.

In relation to the latent subgroups of internet users presenting diminished PIU risk, Demetrovics, Király [28] found a second class that was labelled as ‘*not at risk of PIU*’ (comprising 85.6% of the sample), which was mostly characterized by PIUQ-6-SF items measuring Neglect. This finding is highly consistent with the present findings as our ‘*low PIU risk*’ class was proportionally equivalent in size (i.e., 80%) and similar in terms of the most pronounced PIU symptoms experienced.

In addition to determining and characterizing the latent PIU classes reported in this study, we also sought to compare these classes in order to establish participants’ unique psychosocial risk profiles. Therefore, based on the results obtained, we found partial support to H2. Although participants with membership to the ‘*high PIU risk*’ class had greater levels of PIU (as an overall measure and across its three subfactors), lower subjective well-being, and self-control, our results did not support the expected assumption that ‘*high PIU risk*’ participants would experience lower levels of quality in parent-child relationship. There may be several potential reasons underpinning the lack of a significant relationship between greater PIU risk and lower quality in parent-child relationship.

Firstly, PIU is not yet an officially recognized and psychiatrically accepted addictive disorder. In fact, the notion of ‘Internet addiction’ remains highly controversial and its clinical validity and usefulness has been debated for many years [7,8,64]. While the clinical status of PIU is currently being scientifically challenged, it is clear that if PIU (in its generalized and non-specific form) was to be a *bona fide* addictive disorder, the expected relationships in our cross-class comparisons would likely have been corroborated. Secondly, the psychometric tool used in this study to assess PIU (i.e., PIUQ-6-SF) is an extremely brief tool, and its relatively low number of items may not necessarily provide optimal coverage of the six core components of addictive disorder underpinning higher PIU severity [65].

The relationship between parental relationship and PIU is complex and multifaceted. For example, in a slightly older sample of adolescents, Wang, Li [66] reported that greater quality in parent-adolescent relationship was negatively associated with PIU, supporting the view that a favorable parental relationship may constitute a protective developmental resource capable of buffering problematic behaviors in adolescence. This is because a functional parent-adolescent relationship contributes to the development of emotion regulation skills that can decrease PIU levels [66]. In a recent similar study involving older adolescents, Huang, Hu [67] reported that the quality in the relationship between parent-child was negatively associated to PIU, with this association being mediated by self-concept. Furthermore, in the context of specific PIU problems related to online gaming, among children aged between 12 to 17 years, the quality in parent-child relationship has been reported as a weak correlate of internet gaming problems [68].

The findings of our study present with several important implications to different stakeholders (e.g., caretakers, practitioners, etc.). At the parental level, because high-risk PIU individuals are usually young [69–71], it is important that, beyond employing specific mediation styles, parents work with their adolescent to bolster their levels of emotion regulation as it constitutes a risk factor for PIU [70] exerting indirect effects on problematic usage as evidenced by several studies [66,72]. At the clinical level, if PIU is capable eliciting functional impairments, mental health practitioners should employ clinical interview methodologies and assessment approaches that evaluate adolescents’ mood and time management issues due to their dysregulated internet use as this may provide valuable information about the potential PIU risks and severity of internet-related functional impairments that may be experienced by younger clients.

Moreover, based on the findings obtained in our study and the items mostly predictive of high PIU risk, when it comes to PIU assessment in adolescents, mental health practitioners should pay particular attention to elevated mood-related issues (e.g., withdrawal symptoms, item 2), failure to control the behavior (e.g., internet use dysregulation, item 3), and internet overuse that negatively impacts on quality of life (e.g., sleep problems, item 1) as these were the main features presented by adolescents exhibiting greater risk of PIU in the present study.

The present study also paves the way to future research aiming at expanding the extant knowledge on PIU among youth. Future research may provide valuable contributions to the field by examining how clinically diagnosed PIU individuals compare non-PIU individuals across different psychological and social domains using different analytical approaches such as logistic regression modeling in order to ascertain the probability specific functional impairments may occur across different risk groups.

Despite these promising findings, our results are not free from potential limitations. Firstly, given the large-scale nature of this study and the financial costs involved, we were not able to measure additional factors that may help explain the intricacies between psychosocial factors and PIU risk. Therefore, our findings do not signify the existence of causal relationships based on the results reported as there may be additional underlying mechanisms explaining the relationship between elevated PIU symptoms and greater psychosocial risk. Secondly, the cross-sectional nature of our study hinders the ability to establish directionality for the effects observed even though the relationships reported are likely to take place in a sequenced way. Thirdly, the study would have been able to provide greater insights onto PIU and its associated risks by sampling young internet users from different age groups in order to provide a broader picture of how PIU may unfold across different developmental stages.

Notwithstanding these potential limitations, our findings provide a valuable contribution to the field by helping consolidate the current understanding about the potential impacts of PIU in childhood. The many strengths of this study include the use of a robust analytical and modeling framework to investigate the hypotheses developed, and the use of a large and nationally representative sample of a specific population that remains overlooked in PIU research (i.e., adolescents).

## Conclusion

Based on the findings obtained, it can be concluded that about 20% of Slovenian youth (i.e., eighth graders) are at a greater risk for developing PIU symptoms and related problems. Moreover, greater PIU risk may translate to time management difficulties coupled with mood management issues. The findings obtained further indicated that greater PIU risk was associated with increase PIU symptomatology and reduced levels of subjective well-being and self-control.
